# Regulation of Kv2.1 Channels by Kv9.1 Variants

**DOI:** 10.3390/biomedicines13051119

**Published:** 2025-05-06

**Authors:** Hedaythul Choudhury, Muruj Barri, Kay Osborn, Mohan Rajasekaran, Marina Popova, Owen S. Wells, Edward B. Stevens, Ruth D. Murrell-Lagnado

**Affiliations:** 1 School of Life Sciences, University of Sussex, Brighton BN1 9QG, UKo.wells@sussex.ac.uk (O.S.W.); 2 Metrion Biosciences, Building 2 Granta Centre, Granta Park, Cambridge CB21 6AL, UK; edwardbstevens@gmail.com

**Keywords:** Kv2 potassium channels, pain, neurons, fluorescence imaging, patch clamp electrophysiology

## Abstract

**Background/Objectives:** Kv2 channels have important conducting and nonconducting functions and are regulated by their co-assembly with ‘silent’ Kv subunits, including Kv9.1. Kv9.1 is co-expressed with Kv2 channels in sensory neurons, and a common allele that changes Ile489 to Val in human Kv9.1 is associated with pain hypersensitivity in patients. The mechanism responsible for this association remains unknown, but we hypothesise that these two variants differ in their regulation of Kv2.1 properties, and this is what we set out to test. **Methods:** Expression was carried out using HEK293 cells, OHeLa cells, and primary cultures of hippocampal neurons, and the biophysical and trafficking properties of homomeric and heteromeric channels were assessed by confocal fluorescence microscopy and patch clamp analysis. **Results:** Both Kv9.1Ile and Kv9.1Val were retained within the endoplasmic reticulum when expressed individually, but when co-expressed with Kv2.1, they co-localised with Kv2.1 within the surface clusters. Both variants reduced the surface expression of Kv2.1 channels and the size of channel clusters, with Kv9.1Val producing a greater reduction in surface expression in both the HeLa cells and neurons. They both caused a similar hyperpolarising shift in the voltage dependence of channel activation and inactivation. Concatamers of Kv2.1 and Kv9.1 suggested that both 3:1 and 2:2 ratios of Kv2.1 to Kv9.1 were permitted, although 2:2 resulted in lower surface expression and function. **Conclusions:** The Ile489Val substitution in Kv9.1 does not disrupt its ability to co-assemble with Kv2 channels, nor its effects on the voltage-dependence of channel gating, but it did produce a greater reduction in the Kv2.1 surface expression, suggesting that this underlies its association with pain hypersensitivity.

## 1. Introduction

In recent years, there has been considerable interest in Kv channels as potential therapeutic targets for the treatment of chronic pain [1,2]. Kv channels form a large family, with several different subtypes co-expressed within nociceptive neurons [3,4,5]. These include Kv2.1 and Kv2.2, as well as modulatory or ‘silent’ KvS subunits, such as Kv9.1 and Kv6.4, which do not form functional channels alone but co-assemble with Kv2 subunits to form heteromeric channels [6,7,8]. The dysregulation of Kv2 and KvS subunits has been shown to be associated with altered pain sensitivity [5,9,10,11,12]. For example, in animal models of chronic neuropathic pain, the expression of Kv2.1, Kv2.2, and Kv9.1 channels is down-regulated, and, for Kv9.1, the time course mirrors the development of pain [5,10,11]. Further evidence to support a role for Kv9.1 in modulating pain has come from its selective knockdown in mouse sensory neurons, which caused exaggerated pain responses in models of neuropathy. There have also been single-nucleotide polymorphisms (SNPs) identified in both human Kv9.1 and Kv6.4, which are associated with altered pain sensitivity [13,14]. For example, a rare mutation within the pore region of Kv6.4, Val419Met, which disrupts its ability to form heteromeric channels with Kv2 subunits, produces an increase in the pain threshold and is associated with reduced human labour pain [13]. For Kv9.1, a common SNP that produces a Val substitution for Ile489 was shown to be associated with higher pain scores in five out of six independent patient cohorts and across a number of human chronic pain conditions, including sciatic and phantom limb pain [14]. Additionally, the risk profile was additive, with two copies conferring greater risk than one. The mechanism by which Kv9.1 regulates pain sensitivity remains unknown, but we hypothesise that this occurs via its association with and regulation of Kv2 channels.

KvS comprises a group of 10 subunits that have been identified to date [6]. They share the same architecture as other Kv subunits, where all four subunits of KvS-Kv2 heterotetrameric assemblies contribute to the central conduction pore [7]. The preferred stoichiometry of these assemblies remains controversial. The use of FRET to investigate the channels formed between rat Kv9.3 and Kv2.1 indicated a fixed stoichiometry of one Kv9.3 to three Kv2.1 subunits [15]. A similar structure was proposed for Kv6.4-Kv2.1 heteromeric assemblies based upon both an analysis of the movement of voltage sensors within the channel complex and the counting of subunits within plasma membrane (PM) channels by the bleaching of GFP tags on either Kv6.4 or Kv2.1 [16]. Recent evidence has suggested, however, that the preferred ratio is 2:2, with Kv6.4 and Kv2.1 subunits alternating around the pore [17]. This evidence includes an analysis of the properties of different tetrameric concatamers of Kv2.1 and Kv6.4, and it suggests that more than one stoichiometric arrangement might be permitted, dependent on the KvS subunit involved.

Measurements of KvS-Kv2 channel currents following the expression in heterologous systems indicate that different KvS subunits modulate the conductance properties of Kv2 channels in different ways. Neuronal Kv2 channels are delayed rectifier channels, which play a role in the repolarisation of the membrane potential during action potential firing and regulate the width of the action potentials, the after-hyperpolarisation, and the frequency of firing [18]. The co-expression of Kv6.4 with Kv2.1 produces a small (5 mV) hyperpolarising shift in the voltage dependence of channel activation and a more profound (30 mV) hyperpolarising shift in steady-state inactivation [6,13]. The shift in inactivation and the resultant decrease in the amplitude of Kv currents reduce the threshold current needed to evoke an action potential and hence increase neuronal excitability. Neuronal excitability is predicted to be reduced for the ‘assembly deficient’ Kv6.4 pore mutant, which would explain the elevated pain threshold in individuals expressing this variant. An early study examining the effect of rat Kv9.1 on Kv2.1 currents in Xenopus oocytes also showed a hyperpolarising shift in the voltage dependence of both channel activation and inactivation compared with Kv2.1 alone, as well as a suppression of Kv2.1 current amplitudes [8]. A comparison of how the two human variants of Kv9.1 (Ile 489 and Val489) affect Kv2.1-mediated currents remains to be conducted [14].

In addition to a consideration of the effects of Kv9.1 on the conducting properties of Kv2.1 channels, these channels also have an important non-conducting role, which is to stabilise junctions between the endoplasmic reticulum (ER) and the PM [19,20]. Both Kv2.1 and Kv2.2 form micrometre-sized clusters at the PM, which are thought to comprise hundreds of channels [21,22,23,24,25,26,27]. A clustered distribution for Kv2.1 and Kv2.2 was also recently shown in DRG neurons [28]. Channels within these clusters interact with ER resident VAMP-associated proteins (VAPs), and this is involved in the stabilisation and remodelling of ER-PM junctions [29,30]. This function is independent of the conduction of K^+^ [19] and the channels within clusters are thought to be non-conducting [21,31]. ER-PM junctions are sites of lipid and protein transport [32] and important for Ca^2+^ homeostasis [33,34,35]. In a variety of cells, including pancreatic beta cells and peripheral and central neurons, Kv2.1 clusters have been shown to coincide with insertion platforms for the delivery of Kv2.1 and other ion channels to the plasma membrane, a process dependent upon an interaction between Kv2.1 and syntaxin [32]. Recent studies have also provided evidence for conductance-independent roles for Kv2.1 in regulating Ca^2+^ uptake into the ER and in regulating the release of both dense core vesicles and synaptic vesicles [33,36,37]. How modulatory KvS subunits affect Kv2 channel clusters and the dynamics of ER-PM junctions is unknown. A change in these non-conducting properties of Kv2 would be expected to have an important effect on neuronal transmission.

In this study, we set out to test the hypothesis that the two Kv9.1 variants differ in their regulation of Kv2.1 by examining both the conducting and non-conducting of Kv2.1 in the absence of and then in the presence of either Kv9.1Ile or Kv9.1Val. Although retained within the ER when expressed alone, both variants trafficked with Kv2.1 and Kv2.2 to the PM and co-localised within surface clusters. They produced similar hyperpolarising shifts in the voltage-dependence of Kv2.1 channel activation and inactivation. They decreased Kv2.1 PM expression, and this effect was more pronounced for Kv9.1Val compared to Kv9.1Ile in both HeLa cells and neurons. These results suggest that the link between Kv9.1Val and pain is the inhibition of Kv2.1 PM expression, which is consistent with the downregulation of Kv2 in pain models [5].

## 2. Materials and Methods

### 2.1. Ethics

C57Bl/6J mice were maintained under conventional housing conditions and a 12–12 light–dark cycle. The neonatal mice (postnatal age 0–2 days) used for the experiments were culled by an appropriate Schedule 1 procedure (cervical dislocation) in accordance with the Animals Scientific Procedures Act 1986 amendment regulations 2012. Schedule 1 training of the experimenters was overseen and approved by the local Named Training and Competency Officer (NTCO). Experiments were reviewed a priori by the Animal Welfare Ethical Review Body at the University of Sussex.

### 2.2. HeLa Cell Culture and Transfection

Human cervical carcinoma HeLa cells (obtained from Merck, Dorset, UK, 3021013) were cultured and maintained in Dulbecco’s Modified Eagle Medium (DMEM)/F12 (1:1) (Gibco, Paisley, Scotland, 21331-020) supplemented with 1% GlutaMax (Gibco, Paisley, Scotland, 35050-038), 10% fetal bovine serum (FBS) (Merck, Dorset, UK, 9665), and 1% penicillin/streptomycin (P/S) (Gibco, Paisley, Scotland, 15140122) in 5% CO_2_ at 37 °C. Cells were seeded on coverslips precoated with collagen (Merck, Dorset, UK, 125-50) and grown to a confluency of 40–60%. Cells were transiently transfected 24 h post plating with 1–3 µg plasmids using TransIT-LT1 (Mirus bio, Madison, WI, USA, MIR 2304). Experiments were conducted 48 h post transfection.

### 2.3. Culture and Transfection of Mouse Hippocampal Neurons

To culture hippocampal neurons, 3 to 4 pups of mouse C57BL/6 of either sex on postnatal days 1 to 2 were used to plate 6 wells of a 12-well plate. Briefly, CA1 to CA3 regions with the dentate gyri were dissected in 1×Hank’s Balanced Salt Solution (HBSS) (Gibco, Paisley, Scotland, 14155048) containing 1% P/S and mechanically dissociated by gently pipetting up and down in a plating medium until a homogenous mixture was obtained. The plating media consisted of Minimum Essential Media (MEM) (Gibco, Paisley, Scotland, 51200087) supplemented with 20 mM D-(+)-Glucose (Merck, Dorset, UK, 24895335), 1% P/S, 1% sodium pyruvate, HEPES (Merck, Dorset, UK, H0887), 1×N2 (Gibco, Paisley, Scotland, 17502048), and 10% heat-inactivated horse serum (Gibco, Paisley, Scotland, 26050-088). The mixture was further diluted with the plating media before dispensing 0.5 mL onto 16 mm coverslips pre-coated with poly-D-lysine (50 µg/mL) (Gibco, Paisley, Scotland, A38904-01) and laminin (20 µg/mL) (Merck, Dorset, UK, L2020). Cells were incubated for 2 to 3 h in 5% CO_2_ at 37 °C before adding 2 mL of neurobasal A (Gibco, Paisley, Scotland, 1249015) supplemented with 1×B27 (Gibco, Paisley, Scotland, 11530536), 1% P/S, and 0.5 mM GlutaMAX. Cultures were treated with 3.25 µM of AraC at 4 to 5 days in vitro (DIV). DNA transfection using Ca^2+^ phosphate (Promega, Southampton, UK, E1200) was performed on cultures aged 6 to 7 DIV following a modified protocol described previously (Jiang, M. and Chen, G. 2006 [38]). In our approach, 50 µL of 2×HBS (HEPES buffered saline) and a combined 50 µL solution containing 5 µL of 2 M CaCl_2_, DNA, and H_2_O were employed. Transfected neurons were imaged 48–72 h post-transfection. A minimum of three independent cultures were used to repeat experiments to ensure reproducibility.

### 2.4. HEK293 Cell Culture and Transfection for Electrophysiology and Western Blot

Human kidney embryonic cells (HEK293; a gift from Dr Mark O’Driscoll’s research laboratory at the University of Sussex) were cultured in high-glucose DMEM (ThermoFisher Scientific, Cambridge, UK, 41966-029), supplemented with 1% P/S (Merck, Dorset, UK, P4333), and 10% tet-free FBS (Biosera, Cholet, France, FB1001T). Cells were passaged twice a week and maintained in 5% CO_2_ at 37 °C.

For patch clamp experiments, HEK293 cells were transiently transfected with wild type or mutant human Kv2.1 and human Kv9.1 inserted into the pCDNA5 vector and with EGFP (pMax-EGFP) (Lonza, Slough, UK, VPA-1002) as a transfection marker using the GeneJammer transfection reagent (Agilent Technology, Cambridge, UK, 204130) according to the manufacturer’s instructions. Then, 24 h post transfection (Day 1), HEK293 cells were trypsinised (Gibco, Paisley, Scotland, 253300) and reseeded on poly-D-lysine-coated coverslips (0.1 mg/mL) (Merck, Dorset, UK, 253300) (ThermoFisher Scientific, Cambridge, UK, 11708701) and used for the electrophysiological experiments 48–72 h post-transfection. Only eGFP-positive cells were recorded during electrophysiology studies.

For the Western blot experiments, HEK293 cells were cultured in the normal way, plated in a 6-well plate and transfected 24 h post plating with 2 µg DNA using 2 μL of Lipofectamine 2000 (ThermoFisher Scientific, Cambridge, UK, 11668027) per well.

### 2.5. Preparation of Plasmid Constructs

Human Kv2.1 (GeneCards; *KCNB1* (858aa); NCBI Gene: 3745, OMIM^®®^: 600397, UniProtKB/Swiss-Prot: Q14721); Human Kv9.1 (GeneCards; *KCNS1* (526aa); NCBI Gene (3787), OMIM^®®^: 602905, UniProtKB/Swiss-Prot: Q96KK3); and Human Kv2.2 (GeneCards; *KCNB2* (911aa); NCBI Gene: 3745, OMIM^®®^: 600397, UniProtKB/Swiss-Prot: Q14721) were cloned in the mammalian expression vector pcDNA5, obtained as a gift from our colleague Dr Owen S. Wells, of the Sussex Genome Centre (pcDNA™5/FRT Mammalian Expression Vector) (ThermoFisher Scientific, Cambridge, UK, V601020). EGFP-tagged human Kv2.1 in pEGFP-C1 was obtained from Addgene (Watertown, MA, USA, 111538) and underwent several modifications. A glycine-rich flexible linker (GGGGSx2) was introduced between EGFP and Kv2.1 through polymerase chain reaction (PCR) amplification. The resulting PCR product was then blunt-end ligated using KLD mix (New England Biolabs, Herts, UK, M0554S). A stop codon was added at the end of the Kv2.1 sequence, and a point mutation, Pro129Leu, was introduced to match the sequence of Uniprot (Q14721). The following clones, including FusionRed-Kv2.1, EGFP-Kv2.2, mCherry-Kv9.1(Ile), FusionRed-Kv9.1(Ile), and concatemers, were constructed by Gibson assembly cloning using NEBuilder HiFi DNA Assembly Master Mix (New England Biolabs, Herts, UK, MSS20A) according to the manufacturer’s instructions. All PCRs were performed using the Q5 High-Fidelity Polymerase (New England Biolabs, Herts, UK, M0491S). In brief, FusionRed-Kv2.1 was constructed by replacing the EGFP of the EGFP-Kv2.1 plasmid with FusionRed. EGFP-Kv2.2 was generated by inserting EGFP with the linker sequence at the N terminus of Kv2.2 in pcDNA5. FusionRed-Kv9.1(Ile) and mCherry-Kv9.1(Ile) were made by subcloning both FusionRed and mCherry at the N terminus of Kv9.1 in pcDNA5, respectively. Concatemers were generated by the sequential insertion of individual subunits into Kv2.1 in pEGFP-C1 after removing EGFP. Briefly, EGFP was replaced by Kv9.1 and Kv2.1 in order to make the concatemers Kv9.1-Kv2.1 and Kv2.1-Kv2.1, respectively. The concatemer Kv9.1-Kv2.1-Kv2.1-Kv2.1 was created by subcloning Kv2.1-Kv2.1 downstream of Kv9.1-Kv2.1. The stop codons in between subunits were removed, and the adjacent subunits were segregated by the linker GGGGS×2. The Myc-Kv9.1 clone was created by inserting the Myc tag and linker GGGGS×2 upstream of Kv9.1 in pcDNA5. To generate HA-Kv2.1 and HA-Kv9.1, a single copy of the hemagglutinin (HA) epitope sequence YPYDVPDYA was inserted within the first extracellular domain after glycine 221 and 260, respectively [22]. For both constructs, another glycine was inserted after the HA sequence. The VAPA-EGFP construct was obtained from Addgene (Watertown, MA, USA, 18874). All mutants in this study, including the single nucleotide polymorphism Ile489Val of Kv9.1, Kv9.1(G423S)-Kv2.1, Kv9.1-Kv2.1(G379S) and Kv9.1(G423S) were generated by PCR amplification using either the GeneART (ThermoFisher Scientific, Cambridge, UK, A13310) or the Q5 site-directed mutagenesis kit (New England Biolabs, Herts, UK, M0554S) and mutant primers. Primers, ordered from Merck (Dorset, UK) and used to generate the mutations in this study are listed in Table 1. DNA sequencing by Eurofins Genomics (Ebersberg, Germany) was employed to verify the presence of the desired modification and to ensure the accuracy of the final sequence.

### 2.6. Live Cell Confocal Imaging

For confocal imaging, a coverslip with live cells was mounted into the imaging chamber using the P4 platform and RC-25 chamber (Warner Instruments, Holliston, MA, USA). Cells were incubated in HBSS and imaged at room temperature with a 63×/oil objective and a Leica SP8 confocal microscope using excitation and emission settings as follows: EGFP and Alexa Fluor 488 were both excited with a 488 nm Argona laser, and emission light was detected between 500 and 550 nm. DsRed was excited with a 561 nm DPSS laser, and emission was collected between 580 and 620 nm. FusionRed and mCherry were excited with a 561 nm DPSS laser, and emission was captured between 600 and 640 nm. Far Red 647 was excited with a 633 nm He/Ne laser, and the emission detection range was set between 660 and 700 nm.

### 2.7. Immunostaining and Confocal Imaging

The detection of the cell surface HA-Kv2.1 was performed by incubating live cells for 1 h at 37 °C with anti-HA antibodies (Biolegend, London, UK, 901501) diluted in conditioned media (media previously incubated with cells) at a final concentration of 5 µg/mL. Following antibody incubation, cells were washed twice with prewarmed 1× phosphate buffered saline (PBS) and then fixed with 4% paraformaldehyde. After fixation, cells were washed three times with PBS and maintained in PBS until the secondary antibody application. Alexa Fluor 448 (ThermoFisher Scientific, Cambridge, UK, A11029) or 647 (ThermoFisher Scientific, Cambridge, UK, A21235) conjugated goat anti-mouse IgG were used for secondary detection. Cells were incubated with the secondary antibodies for 1 h at room temperature at a final concentration of 4 µg/mL, followed by three washes with PBS. For imaging, covers lips were mounted either onto microscope slides in Fluoroshield (Merck, Dorset, UK, F6057) or into the imaging chamber (P4 platform and RC-25) and covered in PBS. The fluorescence signal was detected using a Leica SP8 confocal microscope. Gain and laser intensity were kept constant across all groups and experiments to ensure comparable intensity readings and enable quantitative analysis. To avoid potential bias, images used for analysis were randomly selected from different fields of view for each condition.

### 2.8. Image and Statistical Analysis

The analysis of all fluorescence images was carried out using Fiji (1.53i). To measure the surface expression of HA-Kv2.1 in HeLa cells from confocal images, the background was subtracted, and then an ROI was drawn immediately around the cell, and the integrated density was measured per cell. The analysis of confocal images from hippocampal neurons was carried out in a similar way, but this time the ROI was drawn around the soma only. Clusters of HA-Kv2.1 smaller than 0.1 µm^2^ were excluded from the analysis of the mean cluster size.

GraphPad Prism 8.0.2 (GraphPad Software) was used for all statistical analyses of imaging data. Data were log-transformed for a normal distribution. Nested one-way ANOVA was used to compare the groups, followed by Tukey’s analysis for the multiple comparisons test. A nested *t*-test was used to compare the total fluorescence intensity of Kv9.1 variants. Data are presented as mean values ± SEM or with a 95% CI. A *p* value of less than 0.05 was considered statistically significant. Data from HeLa cells were obtained from three independent cultured passages, each derived from separate thawings of the parental cell line, while data from neurons were derived from three independent isolation procedures.

Co-localisation analysis was performed in neurons co-transfected with EGFP-Kv2.1 and mCherry-Kv9.1(Ile) or mCherry-Kv9.1(Val). Images were acquired at a resolution of 1024 × 1024 pixels, with a pixel size of 0.2 × 0.2 µm^2^. Using Fiji (1.53i), ROIs were drawn in the soma, and the Pearson’s correlation coefficient was measured using the JCaOP plugin. This method evaluates the correlation of intensity distributions between the two channels based on the degree of signal overlap on a pixel-by-pixel basis. A nested *t*-test was used to compare the PC between the Kv9.1 variants colocalised with Kv2.1.

### 2.9. Patch-Clamp Electrophysiology

Current recordings were obtained in the whole cell configuration at room temperature (20−23 °C) with an Axopatch−200B amplifier (Axon Instruments, Union City, CA, USA). The current recordings were low−pass filtered through a Bessel filter at 1 kHz, sampled at 1–10 kHz with a Digidata 1550B data acquisition system (Axon™ Digidata^®®^ 1550B Low Noise Data Acquisition System) (Axon Instruments, Union City, CA, USA), and online series resistance compensation was performed. Data storage and command voltages were controlled with the pClamp10.7 software (Axon Instruments, Union City, CA, USA). Online leak current subtraction was carried out with leak currents recorded as a fraction of the current evoked on the opposite polarity. Pipettes were pulled using a P-97 Flaming/Brown Micropipette Puller (Sutter Instruments, Novato, CA, USA) from borosilicate glass capillaries (Warner Instruments, Holliston, MA, USA) and afterward heat polished to obtain patch pipettes with a resistance between 2.5–5 MΩ. The patch pipettes were filled with an intracellular solution (ICS) containing 60 mM KCl, 20 mM KF, 60 mM K-gluconate 1 mM Mg-ATP, 10 mM EGTA, 1.5 mM MgCl_2_, 1 mM CaCl_2_, and 10 mM HEPES, and adjusted to pH 7.32 with KOH (adjusted to 320 mosm). Pipette solutions were prepared in batches, aliquoted, and stored at −20 °C until the day of use. All solutions were equilibrated at room temperature before the experiments were performed. Transfected HEK93 cells were continuously perfused with an extracellular solution (ECS) containing 140 mM NaCl, 5 mM KCl, 2 mM MgCl_2_, 2 mM CaCl_2_, 10 mM HEPES, and 10 mM D-glucose, and adjusted to pH 7.35 with NaOH (adjusted to 300 mosm). Junction potentials between the ICS and ECS were zeroed with the filled pipette in the bath solution. A stock solution of A769662 (MedChemExpress, Cambridge, UK, HY-50662) was made in 100% dimethyl sulfoxide (DMSO) and stored at −20 °C until the day of the experiment. The desired working concentrations of A769662 were made by diluting these stock solutions in ECS solution with a final DMSO concentration of 0.3% and were applied to the bath using a fast perfusion system (RSC200 Rapid Perfusion system and EVH-9 rapid valve system) (BioLogic Science Instruments, Seyssinet-Pariset, France). The recording chamber was grounded directly by an Ag/AgCl pellet. All the chemicals and reagents were purchased from Merck, Dorset, UK.

Voltage protocols were generated using pCLAMP 10.7 software (Axon Instruments, Union City, CA, USA). To generate current families, the voltage was stepped from a holding potential of −90 mV to between −80 mV and +80 in 10 mV steps for either 300 ms or 3000 ms, and this was applied every 10 s (0.1 Hz). Tail currents were measured by stepping back to −40 mV. To measure steady-state inactivation, the voltage was stepped for 30 s from between −80 mV and +25 mV in 15 mV steps. A 300 ms test pulse to +50 mV was applied immediately before and after this, and the peak amplitudes of the currents were compared.

### 2.10. Analysis of Patch Clamp Data

The voltage dependence of activation and inactivation was fitted with a single Boltzmann function according to y = 1/(1 + exp (−(V–V_1/2_)/k)), with *V* representing the voltage applied, *V_0.5_* the voltage at which 50% of the channels are activated or inactivated, and *k* the slope factor. The time constants of activation (from −90 to −20 mV) and deactivation (−40 to −90 mV) were obtained by fitting the raw current traces of either the activation or deactivation protocol with a single or double exponential function. Dose−response curves were obtained by plotting y, the fraction of current modulated at −20 mV, as a function of drug concentration, [D], and fitted with the Hill equation: 1 − y = 1/(1 + (EC_50_/[D])^n^_H_), where EC_50_ is the concentration that generates 50% maximum activation and n_H_ the Hill coefficient. The results are expressed as mean ± S.E.M. GraphPad Prism 10 was used to obtain graphs and perform the statistical analysis. Statistical significance was determined using ordinary one-way ANOVA and Welch ANOVA followed by Dunnett’s multiple comparison test. *p* < 0.05 was considered significant. Data from HEK293 cells were obtained from at least three independent cultured passages of cells.

### 2.11. Western Blot

HEK293 cells were harvested 48 h after transfection and lysed in 20 mM HEPES pH 7.5, 20 mM NaCl, 1 mM MgCl2, and 1% Triton X-100 supplemented with a protease inhibitor (Merck, Dorset, UK, 5892791001), and benzonase (Merck, Dorset, UK, E1014-5KU) at 4 °C for 30 min. Lysates were centrifuged at 13000× *g* for 10 min, and protein concentrations were measured using a BCA assay (ThermoFisher Scientific, Cambridge, UK, 23225) with bovine serum albumin (BSA) as standard. Then, 30 μg of total protein in Laemmli buffer was separated on a 4–12% Bis/Tris Gel (ThermoFisher Scientific, Cambridge, UK, NW04125BOX) and transferred to a nitrocellulose membrane. Concatamers were separated on a 3–8% Tris acetate gel (ThermoFisher Scientific, Cambridge, UK, EA03755BOX). The membrane was blocked with 5% BSA in Tris-buffered saline for 1 h at room temperature and probed overnight at 4 °C with anti Kv2.1 antibody (1 in 2000) (Abcam, Cambridge, UK, Ab192761), anti Myc antibody to detect Myc-Kv9.1 (1 in 1000) (Cell Signalling Technology, Danvers, MA, USA, 2276), and GAPDH (1 in 1000) (Cell Signalling Technology, Danvers, MA, USA, 97166). HRP conjugated secondary antibodies (DAKO, Cambridge, UK, P0447 and P0448) were incubated at room temperature for 1 h to detect proteins. All antibody incubations and washes in Tris-buffered saline with Tween (TBS) between incubations were carried out with rotation. Visualisation was with chemiluminescent reagents (Geneflow, Lichfield, UK, K1-0170). Images were acquired and quantified using GE Healthcare Cytiva ImageQuant 4000, normalising for loading differences with GAPDH [39]. Statistical significance was tested using either a one-way ANOVA with repeated measures or a paired two-tailed *t*-test.

### 2.12. Biotinylation and Streptavidin Pulldown

Cells in a 6-well plate were rinsed twice with ice-cold PBS and transferred to Eppendorf tubes. Cells were incubated with 800 µL of 0.2 mg/mL EZ-Link sulfo-NHS-LC-biotin (ThermoFisher Scientific, Cambridge, UK, A39257) in normal extracellular solution (NES; 140 mM NaCl, 5 mM KCl, 2 mM CaCl2, 1 mM MgCl2, 10 mM D-glucose, 10 mM HEPES, pH 7.3) with rotation for 1 h at 4 °C. Cells were spun for 5 min at 500× *g* and biotin solution was removed; non-reactive biotin was quenched by incubating cells with stock buffer (25 mM Tris-HCl, 150 mM NaCl, 10 mM EDTA, pH 7.5) for 5 min at 4 °C. Cells were washed twice with stock buffer, spinning between washes, then solubilised in 150 µL solubilisation buffer (stock buffer, 1% Triton X-100, 1 mM PMSF, protease inhibitor cocktail). Samples were transferred to Eppendorf tubes and rotated for 30 min at 4 °C, then centrifuged at 16,000× *g*. A 30 µL aliquot of the supernatant was diluted in 4× Laemmli sample buffer and stored at −20 °C as the ‘total’ protein sample, while the remaining supernatant was added to 30 µL of washed Pierce Streptavidin UltraLink Resin (ThermoFisher Scientific, Cambridge, UK, 53113) and rotated for 2 h at 4 °C. Resin was centrifuged at 500× *g* and washed in stock buffer with 1% Triton four times to remove any unbound protein. Proteins were eluted by the addition of 40 µL 1× Laemmli sample buffer and stored at −20 °C. Protein was separated as above with 4 μL of total and 8 μL of biotinylated sample per well.

## 3. Results

### 3.1. Both Kv9.1Ile and Kv9.1Val Co-Localise with Kv2 Channel Clusters at the Plasma Membrane but Reduce Kv2 Surface Expression and Cluster Size

**Figure 1:**
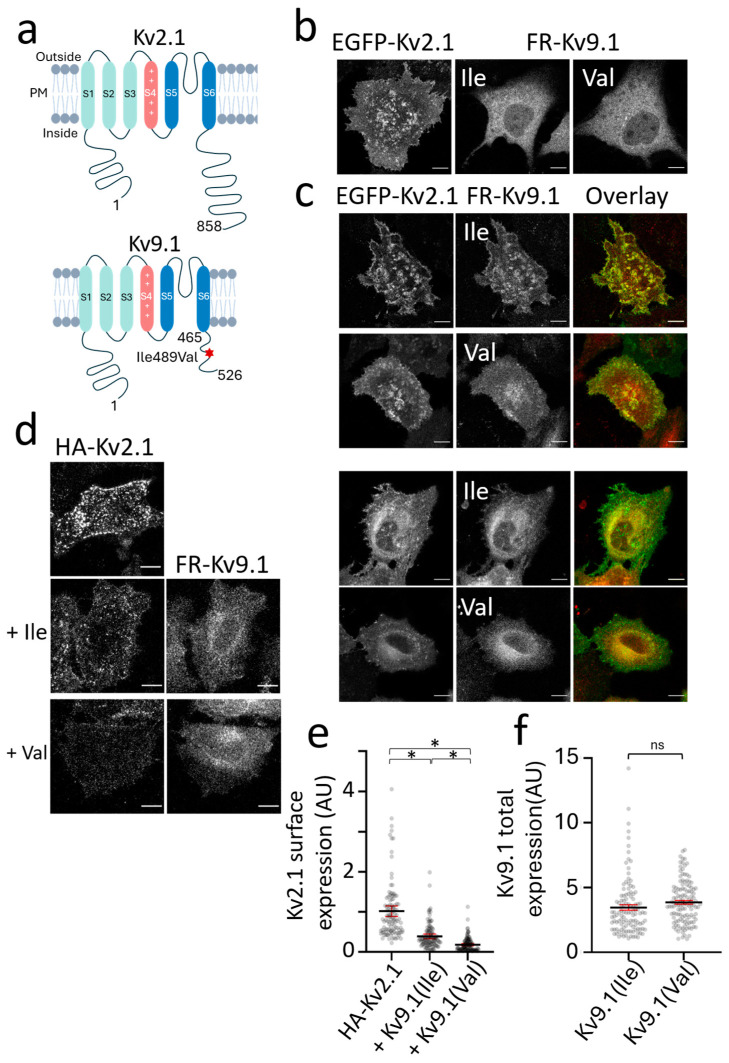
Reduction in Kv2.1 surface expression upon co-expression with either the Kv9.1Ile or Kv9.1Val variant in HeLa cells. (**a**) A graphic depicting the transmembrane topology of human Kv2.1 and Kv9.1, with the position of the Kv9.1 SNP Ile49Val indicated within the middle of the cytoplasmic C-terminal region (*). (**b**) Confocal images of live HeLa cells expressing EGFP-Kv2.1, FR-Kv9.1Ile, and FR-Kv9.1Val. (**c**) Cells in which EGFP-Kv2.1 was co-expressed with either FR-Kv9.1 variant showed two different phenotypes, either increased expression of Kv9.1 at the cell surface and within clusters that overlapped with Kv2.1 clusters (top panels) or increased retention of Kv2.1 within the ER as indicated by the clear outline of the nucleus (bottom panels). (**d**) Surface labelling of HA-Kv2.1 with anti-HA antibody in live cells, when expressed alone (top image) and when co-expressed with either FR-Kv9.1Ile or FR-Kv9.1Val. (**e**) Quantification of the anti-HA immunofluorescence intensity per cell was used to determine the relative surface expression of HA-Kv2.1 when expressed alone (n = 112), together with FR-Kv9.1Ile (n = 115) or together with FR-Kv9.1Val (n = 123); N = 3, three independent passages. The lines represent the mean values with error bars indicating a 95% confidence interval. Nested one-way ANOVA (* *p* < 0.5); by Tukey’s multiple comparisons test. (**f**) In these experiments, the expression of FR-Kv9.1Ile and FR-Kv9.1Val was determined from the intensity of FR fluorescence per cell. Nested *t*-test indicates no significant difference between the two variants. The lines represent the mean with error bars indicating a 95% confidence interval (n = 115 and 123 cells for HA-Kv2.1 plus FR-Kv9.1Ile and HA-Kv2.1 plus FR-Kv9.1Val, respectively; N = 3, three independent passages). All scale bars represent 10 µm. Non-significant results are indicated by ns.

Experiments to compare the effects of the Kv9.1 variants on the subcellular distribution of Kv2 channels were initially carried out using HeLa cells transiently expressing these channels. The topology of Kv2.1 and Kv9.1 is depicted in a schematic, highlighting the Ile489Val SNP located within the C-terminus of Kv9.1 (Figure 1a). Confocal images of Kv2.1 channels, tagged at the N-terminus with EGFP, showed a clustered distribution at the PM similar to previous reports (Figure 1b) [19,22]. By contrast, Kv9.1Ile489 (Kv9.1Ile) and Kv9.1Val489 (Kv9.1Val) had a typical reticular distribution clearly showing the outline of the nucleus that is indicative of localisation within the ER. Upon the co-expression of either the Kv9.1Ile or Kv9.1Val variant with Kv2.1, there were cells that showed a shift in the distribution of these variants to surface clusters that overlapped with the Kv2.1 clusters (Figure 1c, top two rows). There were also cells that showed a change in the distribution of Kv2.1 so that more was evident within the reticular structure surrounding the nucleus (Figure 1c bottom two rows).

To further investigate the effect of the co-expression of Kv9.1 on the PM expression of Kv2.1, we used Kv2.1 with an HA tag inserted within the S1-S2 extracellular loop and the immunolabelled surface exposed Kv2.1 in live cells, with and without Kv9.1Ile and Kv9.1Val (Figure 1d). Surface labelling was reduced by co-expressing either Kv9.1Ile or Kv9.1Val, but there was a significantly greater reduction with Kv9.1Val compared to Kv9.1Ile (5.6-fold versus 2.5-fold, respectively; *p* < 0.0001) (Figure 1e). This effect was not due to differential expression levels of the variants, because the total fluorescence intensity of FR-Kv9.1Ile and FR-Kv9.1Val showed no significant differences (Figure 1f). These results suggest that both Kv9.1Ile and Kv9.1Val can co-assemble with Kv2.1 subunits, resulting in changes in the subcellular distribution of both subunits.

**Figure 2:**
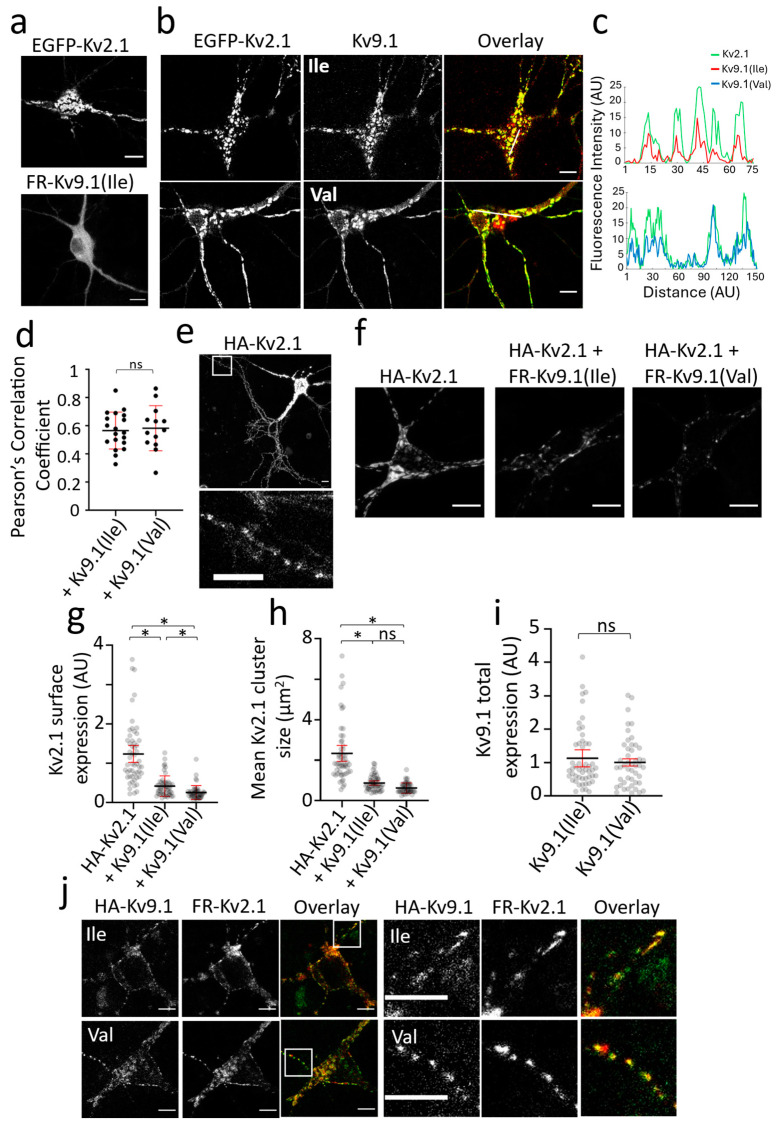
Reduction in Kv2.1 PM expression and cluster size following the co-expression of Kv9.1 variants in hippocampal neurons (**a**) Representative confocal images of live neurons showing the distinct clustering behaviours of EGFP-Kv2.1 in contrast to the diffuse ER distribution of Kv9.1 variants. (**b**) In neurons co-expressing Kv2.1 with Kv9.1, the subcellular distribution of both of the Kv9.1 variants changed and they were present in clusters that overlapped with Kv2.1 clusters. (**c**) Line scan analyses in which fluorescence intensity along the line shown in the overlay images is plotted against distance for Kv2.1 (green), Kv9.1Ile (red), and Kv9.1Val (blue), showing the co-localisation of Kv2.1 and Kv9.1 clusters. (**d**) Pearson’s correlation coefficients were calculated for each Kv9.1 variant co-expressed with Kv2.1 by drawing ROIs within the cell soma. (**e**) Surface immunolabelling of HA-Kv2.1 in live neurons using anti-HA antibody showed high expression at the soma and proximal neurites but also clear punctate distribution throughout distal neurites. White inset box is displayed below, showing a zoomed-in area of distal neurites expressing surface puncta of Kv2.1. (**f**) Representative confocal images show surface labelling of HA-Kv2.1 channels when expressed alone and co-expressed with either FR-Kv9.1Ile or FR-Kv9.1Val. (**g**,**h**) The scatterplots show anti-HA immunofluorescence intensity at the soma of each neuron and the mean size of HA-Kv2.1 clusters at the soma, for HA-Kv2.1 alone and when co-expressed with either Kv9.1Ile or Kv9.1Val. The lines represent the mean with error bars indicating a 95% confidence interval (n = 54, 54 and 51 for HA-Kv2.1 alone, plus Kv9.1Ile and plus Kv9.1Val, respectively; N = 3, independent neuronal isolation procedures). Nested one-way ANOVA (* *p* < 0.05) using Tukey’s multiple comparisons test. (**i**) The fluorescence intensity of FR-Kv9.1(Ile) and FR-Kv9.1(Val) was measured in each cell used in the analysis of HA-Kv2.1 surface expression, and a nested *t*-test indicates no significant difference in the expression of the two variants. The line represents the mean with error bar indicating a 95% confidence interval (n = 54 and 51 cells for HA-Kv2.1 plus FR-Kv9.1Ile and HA-Kv2.1 plus FR-Kv9.1Val, respectively; N = 3, independent neuronal isolation procedures). (**j**) Representative images of anti-HA immunolabelling of HA-Kv9.1Ile and HA-Kv9.1Val co-expressed with FR-Kv2.1 in live neurons. Both variants of Kv9.1 were expressed within surface clusters (green) that overlapped with Kv2.1 clusters (red) both at the soma and within neurites. White inset box is displayed to the right, showing a zoomed-in area of neurites expressing surface clusters of Kv9.1 variants (green) co-localised with Kv2.1 clusters (red). All scale bars represent 10 µm. Non-significant results are indicated by ns.

We next compared the distribution of Kv2.1 and Kv9.1 channels expressed in primary hippocampal cultures to determine if there were differences in channel distribution in post-mitotic cells compared to HeLa cells. Alone, Kv2.1 showed distinct clustering behaviour that was predominantly at the cell soma and in proximal neurites, whereas Kv9.1 expression was diffuse and surrounded the nucleus, consistent with localisation to the ER (Figure 2a). Upon the co-expression of Kv9.1 variants with Kv2.1, the distribution of both Kv9.1 variants became clustered, and these clusters showed a high degree of co-localisation with Kv2.1 at the soma and along the neurites, as revealed by line plots of intensity as a function of distance (Figure 2b,c). As a way of comparing the extent to which the two Kv9.1 variants co-localised with Kv2.1, we calculated Pearson’s Correlation (PC) coefficients for individual cells, and there was no significant difference between the two variants (Figure 2d). To further analyse the PM expression of these channels, we carried out the immunolabelling of HA-Kv2.1 with and without Kv9.1Ile and Kv9.1Val, and the immunolabelling of HA-Kv9.1 variants with and without Kv2.1 in live neurons. Surface clusters of HA-Kv2.1 expressed alone were predominantly at the soma and in proximal neurites, but punctate expression was also apparent throughout the distal processes (Figure 2e). The co-expression of either of the Kv9.1 variants reduced the surface labelling of Kv2.1 and reduced the size of Kv2.1 clusters at the cell soma (Figure 2f–h). Similar to HeLa cells, the Kv9.1Val variant produced a significantly greater reduction in Kv2.1 surface expression compared to the Kv9.1Ile variant (~5-fold and ~3-fold, respectively) (*p* < 0.0001), a difference that was not attributable to a difference in the expression levels of the two variants (Figure 2i). The surface labelling of HA-Kv9.1 variants was not detectable when expressed alone, but both showed punctate labelling in the soma and along neurites when co-expressed with Kv2.1, and these puncta co-localised with EGFP-Kv2.1 (Figure 2j). 

### 3.2. Changes to Kv2 Channel Currents upon Co-Expression with Both Kv9.1 Variants

**Figure 3:**
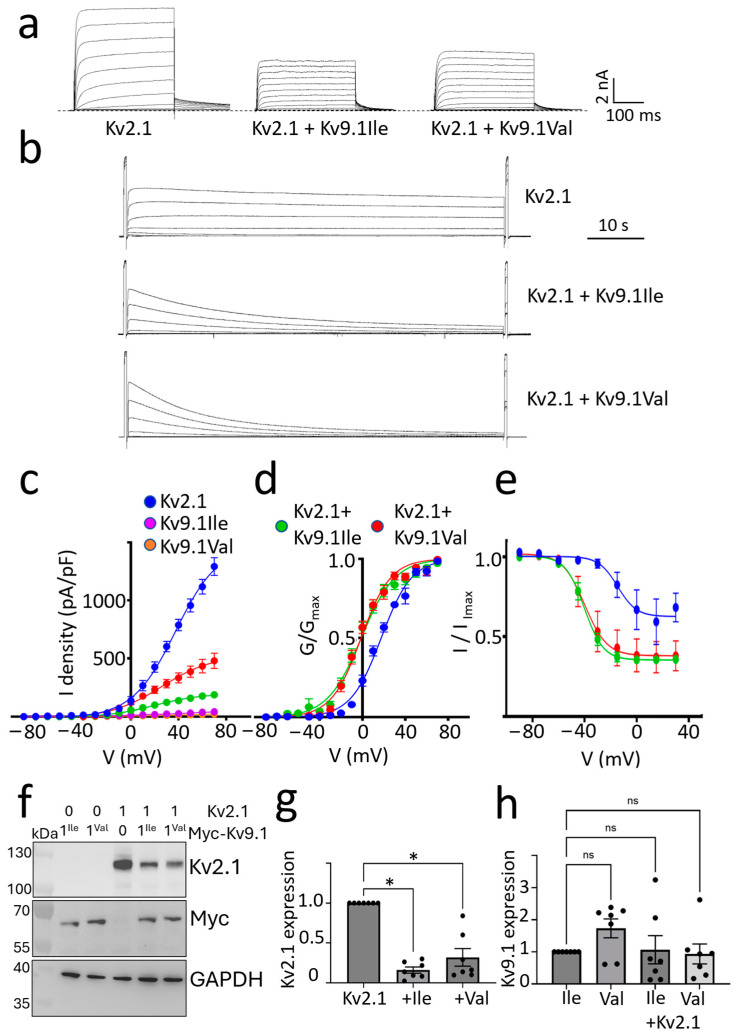
Co-expression of either Kv9.1Ile or Kv9.1Val with Kv2.1, reduced peak current density and Kv2.1 expression and shifted the voltage dependence of current activation and inactivation in the hyperpolarising direction. (**a**) Representative current traces recorded from HEK293 cells transfected with the indicated channel subunits. Currents were evoked by stepping from −100 mV to between −80 mV and +70 mV in 10 mV steps for 300 ms and then back to −40 mV to measure tail currents. (**b**) Representative current traces evoked by stepping the voltage for 30 s from between −80 mV to +25 mV in 15 mV steps. A 300 ms test pulse to +50 mV was applied immediately before and after this, and the peak current amplitudes were compared. (**c**) The mean ± SEM of peak current densities measured at different test voltages using the protocol described in (**a**). Expression of either Kv9.1Ile or Kv9.1Val alone did not give rise to functional channels (n = 3). Co-expression of either Kv9.1 variant with Kv2.1 reduced the peak current density compared to Kv2.1 expressed alone (n = 5 for Kv2.1 alone, n = 11 for Kv2.1 with Kv9.1Ile and n = 14 for Kv2.1 with Kv9.1Val). (**d**) The normalised conductance-voltage relationships with a Boltzmann curve fitted to the data for Kv2.1 alone and Kv2.1 co-expressed with Kv9.1 variants. V50 values (mean ± 95% CI) were 17.6 ± 1.35 mV (n = 5) for Kv2.1 alone, −1.1 ± 1.8 mV (n = 11) for Kv2.1 with Kv9.1Ile and −1.2 ± 1.4 mV (n = 14) for Kv2.1 with Kv9.1Val. (**e**) The voltage dependence of steady-state inactivation measured according to the voltage protocol described in B with Boltzmann curves fitted to the data shows the hyperpolarising shift produced by both variants of Kv9.1. V_50_ values (mean ± 95% CI) were −14.6 ± 11.8 mV (n = 4) for Kv2.1 alone, −39.9 ± 9.1 mV (n = 5) for Kv2.1 with Kv9.1Ile and −40.2 ± 2.8 mV (n = 8) for Kv2.1 with Kv9.1Val. (**f**) Western blot comparing expression of Kv2.1, Kv9.1Ile and Kv9.1Val alone and when co-expressed. (**g**) The mean ± SEM for Kv2.1 corrected for GAPDH expression and then for each experiment normalised to Kv2.1 alone. (**h**) The expression of Kv9.1 was corrected for GAPDH expression, and each experiment was then normalised to Kv9.1Ile alone (n = 4). To test for statistical significance, the non-normalised data were log-transformed for a normal distribution, and statistical significance was tested using a one-way ANOVA with repeated measures followed by Dunnett’s test to assess differences between groups. The reduction in Kv2.1 expression in the presence of either Kv9.1Ile or Kv9.1Val was significant (* *p* < 0.05), whereas there was no significant change in the expression of the Kv9.1 variants in the presence of Kv2.1. Non-significant results are indicated by ns.

The co-expression of KvS subunits with Kv2.1 has previously been shown to shift the voltage dependence of channel activation and inactivation towards more hyperpolarised potentials [6,8]. To compare the effects of Kv9.1Ile and Kv9.1Val on Kv2.1 currents, we expressed hKv2.1 alone or together with either Kv9.1Ile or Kv9.1Val in HEK293 cells; representative current families are shown in Figure 3a,b. Although neither variant was functional alone, both reduced the peak current density when co-expressed with Kv2.1 compared to Kv2.1 alone (Figure 3c). Both variants also produced a hyperpolarising shift in the voltage dependence of channel activation and inactivation compared to Kv2.1 alone (Figure 3d,e). Normalised conductance-voltage relationships were fitted with Boltzmann curves, and V_50_ values (mean ± 95% CI) were 17.6 ± 1.35 mV (n = 5) for Kv2.1 alone, −1.1 ± 1.8 mV (n = 11) for Kv2.1 with Kv9.1Ile and −1.2 ± 1.4 mV (n = 14) for Kv2.1 with Kv9.1Val (Figure 3d). Steady-state inactivation was measured by a short test pulse to +50mV followed by stepping to the indicated voltages for 30 s immediately prior to giving a second test pulse. Boltzmann curves fitted to the data had V_50_ values (mean ± 95% CI) of −14.6 ± 11.8 mV (n = 3) for Kv2.1 alone, −39.9 ± 9.1 mV (n = 5) for Kv2.1 with Kv9.1Ile, and −40.2 ± 2.8 mV (n = 8) for Kv2.1 with Kv9.1Val (Figure 3e). The non-overlapping 95% CIs for these mean V_50_ values comparing Kv2.1 alone with Kv2.1 co-expressed with either of two Kv9.1 variants demonstrate a statistically significant difference in the voltage dependence of activation and steady-state inactivation (*p* < 0.005) [40]. To determine the effects of Kv9.1 variants on Kv2.1 protein expression in HEK293 cells, Western blot analysis was performed. This showed a ~6-fold inhibition of Kv2.1 expression in the presence of either Kv9.1Ile or Kv9.1Val, whereas Kv9.1 expression was not affected by co-expressing Kv2.1 (Figure 3f–h). As a control, we also tested the effects of co-expressing Kv9.1Ile on Kv1.2 expression. These two subunits are not reported to form heteromers, and there was no significant difference in Kv1.2 expression with or without Kv9.1 (Figure S1). These findings indicate that both Kv9.1 variants can assemble with Kv2.1 to form functional heteromeric channels at the PM with altered voltage-dependent gating properties compared to Kv2.1 homomeric channels. The inhibition in the total expression of Kv2.1 suggests either that there is reduced synthesis of Kv2.1 in the presence of Kv9.1 variants or that the assembly of Kv2.1 subunits with either of the Kv9.1 variants accelerates the rate of degradation of Kv2.1 compared to when it forms homomeric assemblies. 

### 3.3. hKv9.1-Kv2.1 Heteromeric Channels with a Stoichiometry of 2:2 (Kv9.1:Kv2.1) Show Increased ER Retention and Reduced Expression and Function

**Figure 4:**
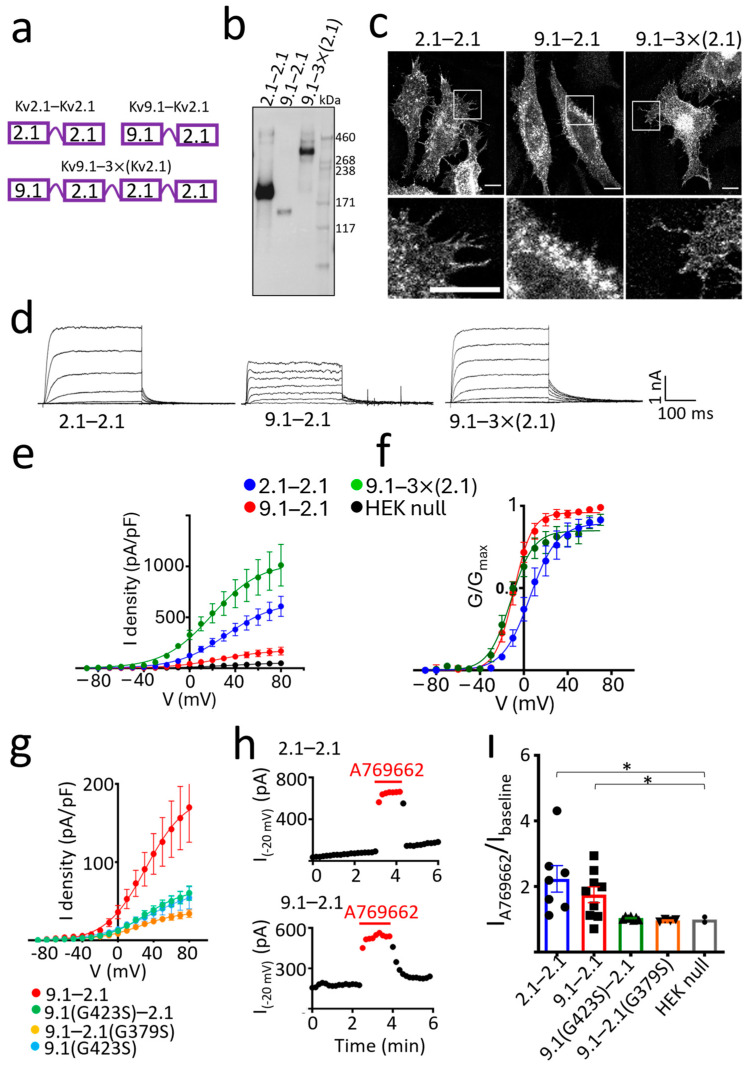
Concatamers of Kv9.1 and Kv2.1 suggest that both a 2:2 and 1:3 stoichiometry is permitted for Kv9.1−Kv2.1 heteromeric complexes. (**a**) Design of dimers comprising Kv2.1 linked to Kv2.1 (2.1−2.1) (top left) and Kv9.1Ile linked to Kv2.1 (9.1−2.1) (top right) and a tetrameric concatemer encoding one Kv9.1Ile linked to three Kv2.1 subunits (9.1−3×(2.1)) (bottom). (**b**) Dimers of 2.1−2.1 and 9.1−2.1 and the tetrameric concatemer 9.1−3×(2.1) were expressed in HEK293 cells, and surface proteins were purified by biotinylation and isolation on streptavidin resin and probed with anti-Kv2.1 antibody following separation on a 3–8% Tris acetate gel. (**c**) Immunostaining with anti-Kv2.1 following expression of the concatamers in HeLa cells showed PM expression for all but greater retention of the 9.1−2.1 dimer within the ER as evidenced by the outline of the nucleus. White inset box is displayed below, showing a zoomed-in area of cells highlighting the PM expression of 2.1−2.1 and 9.1−3×(2.1) and the ER retention of 9.1−2.1. (**d**) Representative whole cell currents recorded from HEK293 cells were evoked by stepping the membrane potential from −100 mV to between −40 mV and +20 mV in 10 mV steps for 300 ms. Tail currents were measured at −40 mV. (**e**) Peak current densities were calculated, and cells expressing the 9.1−2.1 dimer had reduced currents compared to the 2.1−2.1 dimer and 9.1−3×(2.1) tetramer (n = 8 for 2.1−2.1, n = 7 for 9.1−2.1, n = 7 for 9.1−3×(2.1)). (**f**) The hyperpolarising shift in the voltage dependence of channel activation for both 9.1−2.1 and 9.1−3×(2.1) compared to the 2.1−2.1 dimer. V50 values (mean ± 95% CI) were 5.7 ± 4.4 mV (n = 8) for 2.1−2.1, −9.1 ± 1.8 mV (n = 7) for 9.1−2.1, and −11.8 ± 3.0 mV (n = 7) for 9.1−3×(2.1). (**g**) Mutations were made in either the Kv9.1 subunit (G423S) or Kv2.1 subunit (G379S) of the 9.1−2.1 dimer. Both mutations reduced the peak current densities to a level similar to cells expressing Kv9.1(G423S) alone (n = 9 for 9.1−2.1, n = 7 for 9.1G423S-2.1, n = 4 for 9.1−2.1G379S, n = 3 for Kv9.1G423S). (**h**) Currents were evoked by stepping the voltage from a holding potential of −100 mV to −20 mV at 5 s intervals, and peak current amplitudes were measured before, during and after washout of the AMPK activator A769662 (100 µM) and plotted as a function of time. Onset and recovery of the potentiating effect of A769662 were rapid. Results are shown for the 2.1−2.1 dimer (top graph) and the 9.1−2.1 dimer (bottom graph). (**i**) The fold increase in the peak current amplitude in the presence of A769662 is shown for 2.1−2.1, 9.1−2.1 and both mutant 9.1−2.1 dimers, with individual data points shown plus the mean ± SEM. A Welch’s ANOVA was performed, followed by post-hoc comparisons using Dunnett’s T3 test, * *p* < 0.05.

To further investigate the effect of Kv9.1 on the properties of Kv2.1 channels, two concatemers were generated, one with Kv9.1(Ile) linked to Kv2.1 (9.1–2.1) and the other with two Kv2.1 linked together (2.1–2.1) as a control (Figure 4a). WB analysis showed that both constructs produced a band of the predicted molecular mass for their respective dimers (Figure 4b). Confocal images of these two dimers in HeLa cells showed clear PM expression for the 2.1–2.1 dimer highlighted by the filopodia, whereas the 9.1–2.1 dimer showed a clear outline of the nucleus, which is indicative of more expression in the ER (Figure 4c). Whole-cell current densities were lower for 9.1–2.1 and there was a similar ~−15 mV hyperpolarising shift in the conductance-voltage relationship, as obtained with the co-expression of Kv9.1 with Kv2.1 (Figure 4d–f). The 95% CI for the mean V_50_ values were again non-overlapping, indicating a statistically significant difference [40]. Substitution of the Kv9.1 subunit for the Kv2.1 subunit within the dimer constructs appears to either reduce the efficiency of assembly of tetramers or their trafficking to the PM. These results are in good agreement with those obtained with the co-expression of individual subunits, which argues that the Kv9.1 and Kv2.1 subunits behave in a similar way when expressed either as separate entities or when linked together within the same molecule. We also generated a tetrameric construct with one Kv9.1 linked to three Kv2.1 subunits, and although we did not compare this to the tetrameric Kv2.1 construct and therefore cannot make any direct comparison of the current density, this construct gave a band of the predicted molecular mass for this tetramer and also showed clear PM expression and generated large whole-cell currents (Figure 4a–f). These results suggest that heterotetramers with a 1 Kv9.1 to 3 Kv2.1 ratio might traffic more efficiently to the PM than their 2:2 counterparts.

When using concatemers, there is always the concern that the stoichiometry is not as constrained as predicted and that not all subunits linked in the polypeptide contribute to forming the pore. To further examine if the 9.1–2.1 dimers behaved as predicted with both subunits within the dimer contributing to the channel pore, we introduced a mutation within the GYG sequence of the pore region of either the Kv9.1 (G423S) or Kv2.1 (G379S) subunit. Either mutation is expected to disrupt the selectivity filter and inhibit ion permeation if both subunits within the dimer contribute equally to the pore. Neither of the mutant dimers produced a current that was greater than the cells transfected with Kv9.1 (G423S) alone, suggesting that they were incapable of forming channels with a functional pore (Figure 4g). We further tested this by applying the AMP kinase activator A769662 (100 µM), which is known to strongly potentiate rat Kv2.1 currents. This produced a rapid potentiation of 2.1–2.1 and 9.1–2.1 currents measured at −20 mV [41] (Figure 4h,i). In contrast, A769662 had no effect on the current amplitude in cells expressing either of the mutant dimers, 9.1(G423S)-2.1 or 9.1–2.1(G379S), and this was similar to null HEKs (Figure 4i). 

## 4. Discussion

Mouse and human genetic evidence links the sensory neuron Kv2.1 and Kv9.1 channels to chronic pain sensitivity [1,5,10,11,13,14], but the underlying mechanisms remain unclear because Kv2.1 has important non-conducting as well as conducting properties [19,20,29] and Kv9.1 acts as a modulatory subunit with Kv2.1 and Kv2.2 but does not function alone [8]. Validation of these channels as targets for new analgesics requires an improved understanding of how the human variants of Kv9.1 affect Kv2 channel behaviour. Here, we show that both variants of Kv9.1 can assemble with Kv2.1 subunits and locate within surface clusters, but they both act to reduce the surface expression of Kv2.1 and to reduce the size of channel clusters. Both variants altered the biophysical properties of Kv2.1-mediated currents in a similar way, producing a reduction in the peak current density and equivalent hyperpolarising shifts in the voltage dependence of channel activation and inactivation. The suppression of Kv2.1 PM expression was more pronounced for Kv9.1Val compared to Kv9.1Ile in both HeLa cells and hippocampal neurons suggesting that the link between Kv9.1Val and pain is the inhibition of Kv2 PM expression, consistent with reports of downregulation of Kv2.1 in pain models [5].

For the Kv9.1 variants, their suppression of Kv2.1 PM expression was manifested by a reduction in the peak current density along with a reduction in the size of channel clusters. For Kv2.1 channels, a linear relationship between PM expression and current density is not expected, with previous studies showing that only a small proportion of PM Kv2.1 channels conduct K^+^ and that this is also dependent upon channel clustering, with enhanced clustering reducing conduction [31]. The major impact of reducing levels of Kv2 within PM clusters might therefore be upon the structure and function of Kv2-stabilised ER-PM junctions. Given the established role of these junctions in Ca^2+^ homeostatic mechanisms, vesicle secretion, and delivery of ion channels to the cell surface [32,33,36,37], any change in junction stability is expected to impact the transmission of signals from the peripheral pain-sensing neurons.

Our results with the dimer concatamers, showing an inhibition of expression and function combined with the hyperpolarising shift in the voltage dependence of activation upon substituting Kv9.1 for the first Kv2.1 subunit within the dimer, support our findings from co-expression studies and suggest that these are of physiological relevance. The fact that individually expressed subunits behaved similarly to the linked subunits suggests that both variants of Kv9.1 readily associate with Kv2.1 to suppress the number of functional channels at the cell surface. If Kv2.1 subunits displayed a much greater tendency to associate with other Kv2.1 rather than Kv9.1, then we would expect that linking the subunits within the dimer would alter how Kv9.1 affected Kv2.1. channel behaviour. The low PM expression of these complexes suggests either a greater tendency for misfolding and/or the misassembly of 9.1–2.1 heteromers with a 2:2 stoichiometry or greater ER retention of these complexes versus the homotetramers. Our findings with the 9.1–3×(2.1) tetramer need to be interpreted with caution because we did not compare them alongside other tetramers; however, they suggest that the formation of heteromers with a 1:3 ratio of Kv9.1 to Kv2.1 is permitted and that they traffic more efficiently to the PM compared to complexes with a 2:2 ratio. Other studies have also reported that for KvS-Kv2 channels expressed at the PM, the preferred stoichiometry is 1:3 ratio [15,16]. This was shown, for example, for Kv2.1-Kv9.3 channels using FRET analysis [15], and Pisupati et al. reached a similar conclusion for Kv2.1-Kv6.4 channels by measuring the bleaching of GFP-tagged Kv subunits [16]. By contrast, Moller et al. [17] also examined the bleaching of GFP-tagged subunits and combined this with the analysis of dimeric and tetrameric concatemers, and concluded that the preferred stoichiometry of Kv2.1–Kv6.4 complexes was 2:2, although 3 Kv2.1 with 1 Kv6.4 was also permitted.

Several of the KvS subunits have been shown to contribute to physiological and pathophysiological processes, and they share in common their ability to produce a hyperpolarising shift in the voltage dependence of Kv2.1 channel activation and inactivation and to reduce the current density [8,42,43,44,45,46]. The impact of these changes will be dependent upon the firing properties of the cell, with the shift in voltage dependence of channel activation expected to reduce firing frequency and action potential width, whereas the effects on steady-state inactivation and current density are expected to have the opposite effect [6,47]. For the Kv6.4 SNP associated with altered pain sensitivity in human labour, the amino acid substitution within the selectivity filter of the pore disrupts its ability to form a complex with Kv2.1 and reach the PM [13]. Therefore, its modulatory impact upon Kv2.1 channel currents is lost, resulting in a decrease in the firing frequency of sensory neurons, which leads to reduced pain sensitivity. For Kv9.1Ile and Val variants, both were able to traffic from the ER to the PM when co-expressed with Kv2.1 or Kv2.2, and they altered the biophysical properties of Kv2.1 currents in a similar way. The Ile489Val substitution occurs within the middle of the cytoplasmic C-terminal region of Kv9.1 and relatively little is known about how this region affects channel properties. Interestingly, although the key determinant of subfamily-specific assembly of Kv channels is the N-terminal tetramerization domain, for Kv2.1 and Kv6.4 heteromultimers, their subfamily-specific co-assembly was shown to also be dependent upon an interaction between the N-terminus of Kv2.1 and the C-terminus of Kv6.4 [48]. Another KvS subunit that contributes to a pathological process, but this time in mice, is Kv8.2. The Kv8.2 variant with H205 and R252 substituted for R205 and Q252 is associated with an epilepsy phenotype, but both variants form heteromeric assemblies with Kv2.1 and exhibit only minor differences in the heteromeric channel currents, which does not explain the severity of the phenotype [49,50]. The key difference between the two variants appears to be their expression levels within the hippocampus. With mRNA levels for the Kv8.2H205, R252 variant found to be threefold higher, it is thought that this produces an enhanced suppression of the Kv2.1 current density, resulting in increased neuronal firing frequency. Whether or not there are similar differences in the expression of Kv9.1Val versus Kv9.1Ile in human DRG neurons that contribute to the altered activity of these neurons remains to be established.

## 5. Conclusions

We characterised the properties of Kv2.1-Kv9.1 heteromeric channels to better understand the role of Kv9.1 in regulating Kv2.1 channel trafficking and function. Expressed in either HeLa cells or cultures of primary hippocampal neurons, both Ile489 and Val489 variants of human Kv9.1 trafficked with Kv2 channels to the PM and co-localised within surface clusters. Expressed in HEK293 cells, both variants produced similar hyperpolarising shifts in the voltage dependence of Kv2.1 channel gating. Observed in all these cell types was the effect of Kv9.1Val and Kv9.1Ile on Kv2.1 expression. Both variants produced a considerable reduction in Kv2.1 PM expression, and, given that this was more pronounced for Kv9.1Val compared to Kv9.1Ile, it suggests that the link between Kv9.1Val and pain is the inhibition of Kv2 PM expression, consistent with the downregulation of Kv2 in pain models [5].

## Figures and Tables

**Table 1:** Oligonucleotides used in this study. Lower case letters represent mutations.

Name	Sequence (5′-3′)
Pro129Leu-Kv2.1-F	TCGACGAGATCTACCtGGAGTCCTGCTGCC
Pro129Leu-Kv2.1-R	GGCAGCAGGACTCCaGGTAGATCTCGTCGA
Stop-Codon-Kv2.1-F	ACGAGATCAGAGCATCtaGGTACCGCGGGCCC
Stop-Codon-Kv2.1-R	GGGCCCGCGGTACCtaGATGCTCTGATCTCGT
Kv2.1-G379S-F	CATGACTACTGTTaGtTATGGAGACATCTACC
Kv2.1-G379S-R	GGTAGATGTCTCCATAaCtAACAGTAGTCATG
Kv9.1-G423S-F	CATGACCACCGTGaGCTATGGGGATGTGGTGC
Kv9.1-G423S-R	GCACCACATCCCCATAGCtCACGGTGGTCATG
Kv9.1-Val-F	TTGATGGGGTGTCGGAGGCAT
Kv9.1-Val-R	cGCTGCTCAGCAAGTCCTCAAACTC

## Data Availability

The original contributions presented in this study are included in the article/Supplementary Materials. Further inquiries can be directed to the corresponding author.

## Supplementary Materials

The following supporting information can be downloaded at: https://www.mdpi.com/article/10.3390/biomedicines13051119/s1, Figure S1. Western blot comparing expression of Kv1.2 and Kv9.1Ile alone and when coexpressed, shows no change in Kv1.2 expression in the presence of Kv9.1Ile. The graph shows mean ± SD (n = 3) for Kv1.2 expression in the presence of Kv9.1Ile and this was corrected to GADPH expression and for each experiment this was then normalized to Kv1.2 alone. A paired two-tailed *t*-test was performed on the non-normalized data and it failed to show significance.

## Abbreviations

The following abbreviations are used in this manuscript:

BSA	Bovine Serum Albumin
DIV	Days in vitro
DMSO	Dimethyl Sulfoxide
ER	Endoplasmic Reticulum
ECS	Extracellular Solution
FRET	Foster resonance energy transfer
FR	Fusion Red
HBS	HEPES buffered saline
HBSS	Hank’s Balanced Salt Solution
hKv	Human voltage-gated potassium
ICS	Intracellular Solution
Ile	Isoleucine
Kv	Voltage-gated potassium
KvS	Electrically silent voltage-gated potassium
PBS	Phosphate buffered saline
PC	Pearson’s correlation
PCR	Polymerase chain reaction
PM	Plasma membrane
P/S	Penicillin/streptomycin
SNP	Single nucleotide polymorphism
Val	Valine
VAMP	Vesicle-associated membrane protein
