# Supplementary material for: Regulation of Kv2.1 Channels by Kv9.1 Variants
Source: Biomedicines. 2025 May 6;13(5):1119. doi: 10.3390/biomedicines13051119 (PMC12108608; doi:10.3390/biomedicines13051119)
Supplement: Supplementary file 1 [file biomedicines-13-01119-s001.zip › biomedicines-3499311-supplementary.pdf]

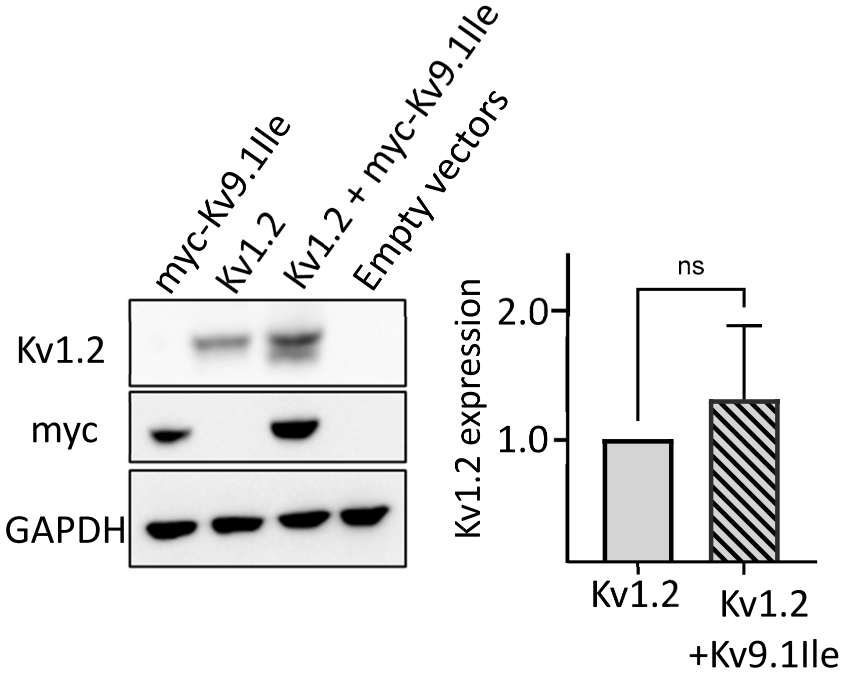

Supplementary Figure S1. Western blot comparing expression of Kv1.2 and Kv9.1Ile alone and when coexpressed, shows no change in Kv1.2 expression in the presence of Kv9.1Ile. The graph shows mean ± SD (n = 3) for Kv1.2 expression in the presence of Kv9.1Ile and this was corrected to GADPH expression and for each experiment this was then normalized to Kv1.2 alone. A one-way ANOVA with repeated measures was performed on the non-normalized data and it failed to show significance.
